# Application of Spatial Cues and Optical Distortions as Augmentations during Virtual Reality (VR) Gaming: The Multifaceted Effects of Assistance for Eccentric Viewing Training

**DOI:** 10.3390/ijerph19159571

**Published:** 2022-08-04

**Authors:** Alexandra Sipatchin, Miguel García García, Yannick Sauer, Siegfried Wahl

**Affiliations:** 1 Institute for Ophthalmic Research, 72076 Tübingen, Germany; 2 Carl Zeiss Vision International GmbH, 73430 Aalen, Germany

**Keywords:** AMD, eccentric viewing training, virtual reality (VR), gaming, augmentation, salience

## Abstract

The present study investigates the effects of peripheral spatial cues and optically distorting augmentations over eccentric vision mechanisms in normally sighted participants with simulated scotoma. Five different augmentations were tested inside a virtual reality (VR)-gaming environment. Three were monocular spatial cues, and two were binocular optical distortions. Each was divided into three conditions: baseline with normal viewing, augmentation with one of the assistance methods positioned around the scotoma, and one with only the simulated central scotoma. The study found that the gaming scenario induced eccentric viewing for the cued augmentation groups, even when the peripheral assistance was removed, while for the optical distortions group, the eccentric behavior disappeared after the augmentation removal. Additionally, an upwards directionality of gaze relative to target during regular gaming was found. The bias was maintained and implemented during and after the cued augmentations but not after the distorted ones. The results suggest that monocular peripheral cues could be better candidates for implementing eccentric viewing training in patients. At the same time, it showed that optical distortions might disrupt such behavior. Such results are noteworthy since distortions such as zoom are known to help patients with macular degeneration see targets of interest.

## 1. Introduction

The macula is a small region of the retina (∼5.5 mm in diameter) responsible for depicting 4% of our visual field [1,2], an even smaller part (∼2 mm), the fovea, accounts for about 1% of our visual field [2]. However, the fovea region is the only part of the visual field that allows for the highest visual acuity. It is through it that most of the usual information for daily activities such as reading, driving, and face recognition is acquired [3]. Objects appearing outside this area are also perceived, but there is a constant re-alignment of the eye with the objects of visual focus to resolve them in detail. It is referred to as the fovea-based oculomotor routine [4].

Damage to the macula due to local or entire degeneration leads to a form of central visual loss referred to as scotoma. This visual loss ultimately disrupts the foveal mechanism [2]. Macula’s alteration has a highly detrimental impact on the patients’ lives, considering its importance for daily life. The prevalence of this condition is steadily increasing [5], with an estimation that by 2040, it will reach pandemic proportions [6], with 288 million people afflicted [7].

Therefore, considering the burden of this condition, it is a pressing matter for the health community to find ways to help patients continue with their daily activities. In general, patients suffering from macular degeneration macular degeneration (MD) can continue being functional individuals as their peripheral visual area remains unaffected by the disease. This area can be used to look at objects. Both patients and even participants with simulated central scotoma [8,9,10,11], use an adaptive mechanism where both groups gaze away from the target of interest. They use their peripheral vision to examine the targets by placing the scotoma away from the region of interest. This behavior is called eccentric viewing. Furthermore, the peripheral visual area constantly aligning with the target is referred to as the preferred retinal locus (PRL) [12]. The PRL becomes the new oculomotor reference in the visual field.

When developing the PRL, it is crucial to carefully choose a part of the intact peripheral vision that can maximize visual performance by being the closest to the damaged area and having the highest acuity. The closer the target is to the scotoma, the higher the sharpness and the better the chances of detecting that object’s properties. Peripheral vision is known to have a minor visual quality and less of a one-on-one representation of the outside world the further away it is placed from the macula [13]. A correct adaptation to this mechanism allows patients to still detect the needed features of an object during daily activities. Unfortunately, not everyone adapts to it, and some may have an inefficient PRL because it is not far enough or too far away from the affected central vision [2]. Therefore, it is essential to help patients to develop an appropriate PRL by presenting effective assistive training techniques.

Thanks to recent technological advances, extended reality (XR) technology, which includes virtual and augmented reality, has seen a rapid increase in its use as a rehabilitation technique to assist low vision patients [14,15,16,17,18,19].

Research has shown that peripheral stimuli can influence salience, or where the current focus of visual attention is allocated while viewing eccentrically [20,21,22,23]. Assistive tools for patients with macular degeneration have been developed as spatial cueing, meaning attention was drawn to a specific location, and as optical distortions in the periphery in combination with virtual reality [24,25,26,27].

Investigating the impact of gaze-contingent peripheral stimuli is essential, considering that information presented in the periphery can affect eccentric viewing. This study aims to test the gazing behavior under the presentation of gaze-contingent peripheral stimuli in the presence of a simulated scotoma. Few studies have investigated how eccentric viewing behavior is affected by different types of stimuli presented in the periphery when a scotoma is present. The current study used different peripheral stimuli in virtual reality to investigate their effects on eccentric viewing training in participants with a gaze-contingent simulated central scotoma. A gaming environment was used to stimulate target tracking.

Five stimuli were tested: three without and two containing distortions. Three were monocular cues applied to the dominant eye as an eye-of-origin singleton stimulus. An eye-of-origin singleton stimulus is known to induce saliency, a type of attention, where the eyes automatically be drawn to [28]. The monocular augmentations were a concentric ring, known to induce salience effectively [24] and an annular area either blurred in or out around the scotoma. Several studies have evidenced that blur applied as a singleton efficiently captures attention [29,30,31]. The other two augmentations were zoom and fish-eye distortions presented binocularly. Both have been investigated previously as assisting viewing aids [32,33].

In this study, a two-dimensional virtual reality gaming environment was used. Training with virtual reality gamification applications has been substituting more traditional rehabilitation techniques thanks to the engagement and learning effect these technologies offer to patients. Recent surveys have shown that rehabilitation with gamification techniques stimulates reward-related systems in the brain known to facilitate learning [34]. Additionally, it has been shown that gaming in combination with VR offered the advantage of reducing the complexity of a task [35]. Furthermore, combining VR, augmentation, and gamification expanded use frequency [36]. All these factors are necessary features for applications developed for training and extensive use, such as training for eccentric viewing. In the present context, gamification in extended reality was presented to train eccentric viewing.

## 2. Materials and Methods

### 2.1. Participants

Thirty participants participated in the study (with a gender balance ratio of 15 females and 15 males, mean age 27, standard deviation (SD) ± 4 years, with only one subject wearing eye correction: contact lenses). Seventeen participants had a previous experience with a virtual reality headset.

### 2.2. Set-Up

For the design of the virtual experiment, the rendering engine Unity 2019.3.0, together with the programming language C#, was used. The experiment ran on a desktop PC with Windows 10 Home, having a 64-bit operating system, an Intel Core i5-7500 processor with 3.41 GHz, and random-access memory (RAM) of 16 GB. A NVIDIA GeForce RTX 3080 Ti was used as a graphics card.

The HTC Vive Pro Eye [37] headset was used to present the virtual environment. The headset has an integrated eye tracker with a sampling frequency of 120 Hz and a known latency between 58 ms and 80 ms [38,39]. The HMD has two AMOLED screens, each with a resolution of 1.440 × 1.600 pixels, meaning a pixel density of 615 pixels per inch (PPI) for each eye, a field of view of 94${}^{\circ }$× 91${}^{\circ }$ (horizontally per vertically) [40], a luminance of 116.1 cd m^−2^ [40] and a refresh rate of 90 Hz. The Tobii Pro SDK v1.7.1.1081 [41] was used to save the eye-tracking data. The Vive SRanipal SDK v1.0.3.0 [42] was used for the gaze-contingent scotoma simulation. An Xbox Microsoft wireless controller was used for the subject’s input.

### 2.3. Calibration Procedure and Manual Drift Correction

An inter-pupillary distance (IPD) adjustment and a five point calibration of the eye tracking (SRAnipal), were performed for all participants before the measuring phase started [24]. After calibration and IPD adjustment, subjects could begin with each condition type. At the beginning of each block of each condition type, a manual drift correction was carried out [24]. Each block started after the experimenter checked the accuracy of the manual drift correction.

### 2.4. Experimental Procedure

Before the experiment started all participants had a first initial trial out session of the VR gaming environment until they felt confident with it. Afterwards participants were asked to play the 2D VR Pong game [24] consisting of a 3${}^{\circ }$ ball moving at an average velocity of 21.74 ${}^{\circ }$ s^−1^ (SD: ±0.63 ${}^{\circ }$ s^−1^) and two paddles at the left and right edge of the play area. Whenever the ball hit one of the paddles, it bounced off with the same speed again and moved on straight lines in different (random) directions. The subjects could control the two paddles to maintain the ball inside the play area. Participants had to restart the game if the ball exited the play area.

Additionally, to motivate patients to track the target eccentrically, they were asked to press a button whenever the ball’s color changed. The ball changed color at random intervals, and correct identification of color change awarded them points. A time of 2 s was provided to indicate the color change. Moreover, a concentric rectangle, head-fixed, measuring ± 14.25${}^{\circ }$ horizontally and vertically, was used to motivate the subjects to move their heads.

#### 2.4.1. Conditions

**Figure 1:**
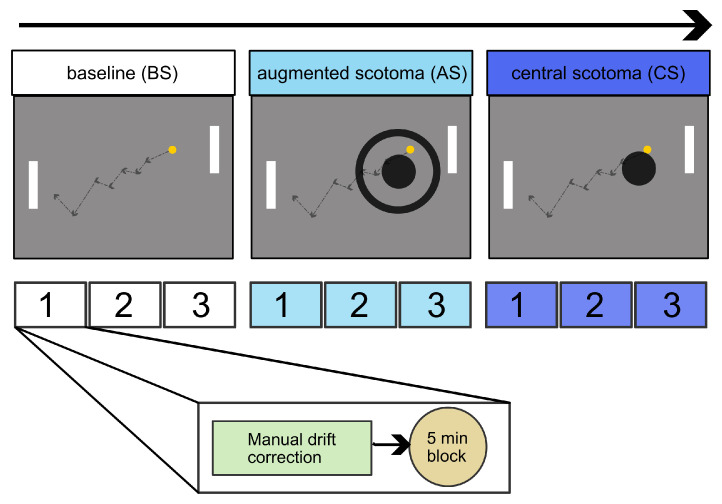
Diagram flow of the study. First, the participants played a baseline (BS) game condition using their normal vision. During gaming, the ball followed a random trajectory (dotted line). Afterward, the augmented scotoma (AS) was presented, showing either a monocular cue or binocular distortion augmentation around the scotoma. The current figure showcases the monocular ring augmentation. The last condition was the central scotoma simulation (CS). Each condition has three repetitions (1, 2, and 3) containing the manual drift correction and a 5 min block.

To each participant, three conditions were presented in the following order (Figure 1): baseline (BS), augmented scotoma (AS), and central scotoma (CS). During the first condition, no simulation nor augmentation was applied to play the game. In the augmentation condition, a monocular or binocular peripheral assistance was presented around a binocular scotoma simulation of 12${}^{\circ }$. Both augmentation and binocular scotoma were gaze-contingent. For the third condition, only the gaze-contingent binocular scotoma simulation was used to play the game.

Each condition was (15) minutes long and divided into three gaming blocks of 5 min. After each gaming block, there was a break during which subjects had to perform the manual drift correction; after the break, the following block started.

#### 2.4.2. Groups

**Figure 2:**
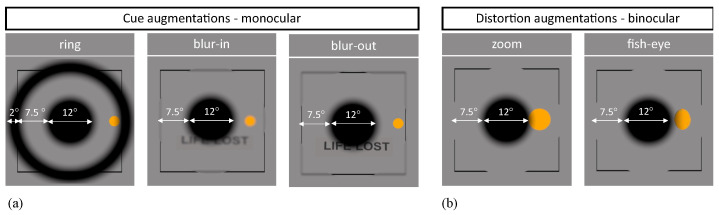
Screenshots and specifications of the monocular (**a**) and binocular augmentations (**b**) as they looked in the VR environment and the different impacts they had on the target. The big black circle is the gaze-contingent scotoma simulation, and the yellow one is the target: the ball. A text is added to show better the bluring effect for the blur-in and blur-out augmentations.

Participants were divided into five groups depending on the type of assistance presented during the augmented scotoma condition. The five types of augmentations were three cued and monocular and two binocular and with optical distortions. The monocular ones were applied to the dominant eye and concentrically around the scotoma: one was a 2${}^{\circ }$ broad black ring, 7.5${}^{\circ }$ away from the simulated scotoma’s edges and the other two were an area of 7.5${}^{\circ }$ diameter, either blurred in or out (Figure 2a: ring, blur-in, blur-out). The blur had a kernel size of 20 pixels and a sigma value of 5. These values were found to induce changes in gaze directionality when used peripherally [43,44]. The binocular optical distortion augmentations displayed were zoom and fish-eye (Figure 2b: zoom and fish-eye). The magnification applied was 2.11 both for height and width, considered to be a good value and not above a multiple of four as indicated by Goldstein et al. [45,46]. The distortion applied had a power of 2.11 on the height and 1.66 on the width following [33].

## 3. Data Processing

### 3.1. Color Change Task: Decoding Where the Scotoma Was Displayed

Gaze-contingent paradigms are lag sensitive. When presenting certain features based on the position recorded by an eye tracker, there is always a delay between the registered location and the displaying of the wanted feature [47]. For the current study, having such a delay meant that the subject could have seen parts or the entire target outside the designed radius of the simulated scotoma. Such latency fault was causing an error in the correct vs. incorrect recognition task ratio.

Therefore, the subjects’ eye position during the color change throughout the scotoma condition was considered. First, Bayes’s theorem was used, and the positive and negative predictive values were calculated at different gaze-target distances in the same manner as Sipatchin et al. [24]. A latency error in rendering the central scotoma would be indicated by a sudden increase in the positive predictive value and a decrease in the negative predictive value. The gaze-target distances where this would be observed represented the point from where the scotoma edge was. Additionally, as a further control, the fixation frequency during the time given to respond to the color change was considered. For this analysis, the non-smoothed gaze-target distance data was used. A fixation was defined as the gaze-target distance that was within 1${}^{\circ }$ and kept long enough to process visual information (between 100ms–250ms) [48,49]. The fixation frequency was calculated as the total number of fixations made per second during the time interval subjects could indicate the correct color change. Literature has shown that simple features require one or two eye fixations to be recognized [50,51,52]. Fixations above two indicate further extraction of visual detail [53,54]. Therefore, a fixation frequency between one and two indicated efficient and rapid information processing. At the same time, further increases above two indicated inefficient processing. Goldberg and Kotval [55] have indeed shown that an increase in fixation frequency translated into a more inefficient search to find the relevant and needed information. During the color change task, different scotoma radii from 7${}^{\circ }$ to 3${}^{\circ }$, in steps of 1${}^{\circ }$ were tested to identify where would have been detected a fixation rate > 2. If the fixation rate was >2, the eyes needed more fixations because the target could not be perceived immediately, indicating an obstacle and, therefore, the presence of the scotoma.

These two tests additionally allowed for the identification of outliers. Each subject that was not in line with the majority trend and did not show a sudden increase and decrease in the positive and negative predictive value, respectively, and/or a fixation frequency above two was considered an outlier.

### 3.2. Data Analysis

The eye-tracking data first underwent a pre-processing phase. A noise cancellation filter [56,57], a latency error correction, and saccades smoothing [58] were applied as described in Sipatchin et al. [24].

#### 3.2.1. Gaze-Target Distancing: Influence of Condition Type over the Gaze Distance

Following the pre-processing step, the normality of the data was then tested with the Kolmogorov–Smirnov one-sample test (*p* < 0.001). The data was not normally distributed; therefore, the non-parametric Kruskal–Wallis test was used to test the effect of the condition type (independent variable) over the median distance between the gaze and the center of the target (dependent variable). These results were further processed using a post hoc FWER test (Dunn–Šidák).

#### 3.2.2. Gaze Position in Relation to Target: Directional Change across Conditions

Circular statistics [59] were used to identify changes in gaze position relative to target across the three different conditions between the five groups. First, the data were checked for circular uniformity using the Rayleigh test. The data was a non-uniform distribution; therefore, the inverse Power Batschelet distribution was used to analyze the gaze position relative to the target. More specifically, the modified version of the inverse Power Batschelet with application to eye data was used [60,61]. Four different functions were used to fit the data which represented the four peaks (close to right, left, up and down) [60]. All exemplified directions were where the gaze could have been directed with respect to the target. The gaze direction in relation to the target was plotted on a polar histogram, and the data were fitted using a mixture of the four circular distributions, one for each direction. A non-linear fit square model was used to fit the data, and an R-squared and root mean square error (RMSE) were calculated to identify the model’s goodness of fit. For each of the distributions, the fitting function $f\left(\theta |\mu ,\kappa ,\lambda \right)$ given in Equation (1) was used.

(1) $$\begin{array}{c} \begin{array}{cc} f\left(\theta |\mu ,\kappa ,\lambda \right) & =\frac{1}{N}t\left(\theta |\mu ,\kappa ,\lambda \right)\\ t\left(\theta |\mu ,\kappa ,\lambda \right) & =\exp\left[\kappa \cos\left(\mathrm{sign}\left(\theta -\mu \right)\pi {\left(\frac{\left|\theta -\mu \right|}{\pi }\right)}^{\frac{1-0.405228\lambda }{1+0.405228\lambda }}\right)\right] \end{array}\; \end{array}$$

where $N=\int_{-\pi }^{\pi }t\left(\theta |\mu ,\kappa ,\lambda \right)d\theta$ is a normalisation constant and κ and μ representing, respectively, the concentration and the peak position parameter; λ as the peakedness adapted from the Jones–Pewsey distribution [62] and the Inverse Batschelet distribution [63]. All parameters were normalized across the four distributions.

Each function was summed up with different weights ω constrained to the sum of one since the mixture model of the four directions lies on a simplex. The ω is used as an indication of a directionality bias, and it quantifies the relative importance of all parameters [60,61].

## 4. Results

### 4.1. Color Change Task: Decoding Where the Scotoma Was Displayed

**Figure 3:**
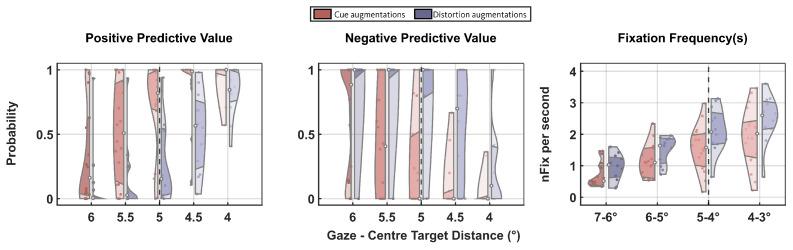
Violin scatter plots for positive and negative predictive values and fixation frequency of all subjects across different distances between gaze and target separated for the cued (red) and optical distortions (blue) augmentations. The black dotted line placed at the value 5${}^{\circ }$ inside the plots indicates the point from which a plateau is starting to emerge for the positive and negative predictive values when considering the monocular augmentations. Furthermore, for the fixation frequency shows where the fixation frequency remains within the two fixations per second range. The plateau and the increase in fixation frequency are starting to emerge below 5${}^{\circ }$.

As previously [24], for the cued assistive methods (red, Figure 3), the positive and the negative predictive values reached a plateau and evidenced a sudden increase and decrease, respectively, at 5${}^{\circ }$ distance between gaze and target. Additionally, it was observed that a fixation frequency bigger than two for values between 5${}^{\circ }$ to 3${}^{\circ }$.

During the optical distortions, the positive and negative predictive values did not reach a plateau at 5${}^{\circ }$ distance between gaze and target, but it was sill observed a high number of fixations (Fixation Frequency(s), blue, Figure 3) whenever the gaze-target distance was between 5${}^{\circ }$ to 3${}^{\circ }$.

Positive and negative predictive values and fixation frequencies showed that some subjects performed the task correctly across all conditions. In contrast, others did not, as evidenced by the violin plots skewed at the edges. For the monocular augmentations, the probability of positive and negative predictive values and the fixation frequency were used to detect divergence from the trends across subjects. At the same time, only the fixation frequencies indicated subjective deviation for the distortions. A total of 6 subjects were excluded, one per each augmentation type; therefore, for the analysis, 25 participants were included, five from each group.

### 4.2. Data Analysis

#### 4.2.1. Gaze-Target Distancing: Influence of Condition Type over the Gaze Distance

**Figure 4:**
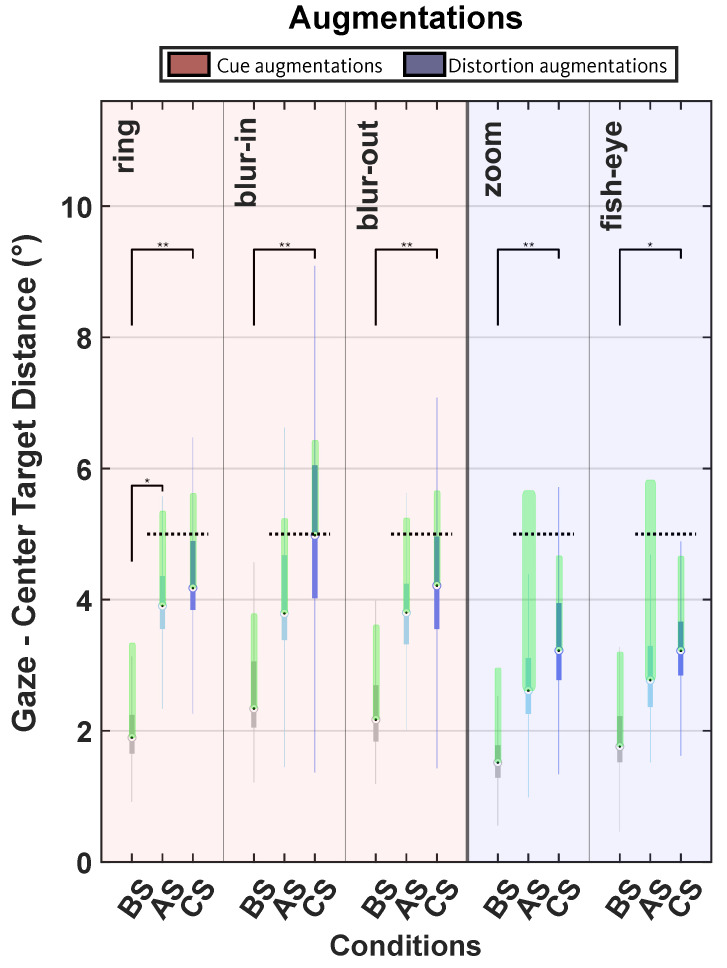
Box plots of the distance between gaze and target center for all the augmentations and all the three conditions (baseline (BS), augmented scotoma (AS), and central scotoma (CS)) tested. The asterisks above the box plots indicate the post hoc Dunn *p*-values, one for *p*-values ≤ 0.05 and two for *p*-values ≤ 0.01. The green rectangles represent the target radius, and the dotted lines are where the edge of the scotoma was considering the latency error. The target is distorted for the zoom and fish-eye during the augmentation condition. Zoom induced a 2.11 multiplication factor on both height and width, while the fish-eye generated a magnification of 2.11 on height and 1.66 on width.

Both monocular and binocular augmentations induced a significant change in gaze-target distance between conditions (ring: $\chi^{2}$ (2) = 9.50, *p* = 0.01; blur-in: $\chi^{2}$ (2) = 8.54, *p* = 0.01; blur-out: $\chi^{2}$ (2) = 9.78, p = 0.01; zoom: $\chi^{2}$ (2) = 10.50, *p* = 0.01; fish-eye: $\chi^{2}$ (2) = 7.74, *p* = 0.02). The post hoc Dunn test evidenced that the scotoma condition was significantly different than the normal condition following all augmentations: the ring (*p* = 0.01), blur-out (*p* = 0.01), blur-in (*p* = 0.01), zoom, (*p* = 0.01), and the fish-eye (*p* = 0.02) augmentation; and only the ring augmentation induced a significant change following the normal condition (ring, *p* = 0.04). The monocular augmentations were observed to induce an eccentric viewing behavior that was preserved during the scotoma condition, this was not the case for the binocular ones (Figure 4).

#### 4.2.2. Gaze Position in Relation to Target: Directional Change across Conditions

There was adequate goodness of fit for all augmentations and across all conditions (R-squared ≥ 0.94, RMSE = 0.01). The four parameters evidenced changes in the circular distributions across the three different conditions.

**Figure 5:**
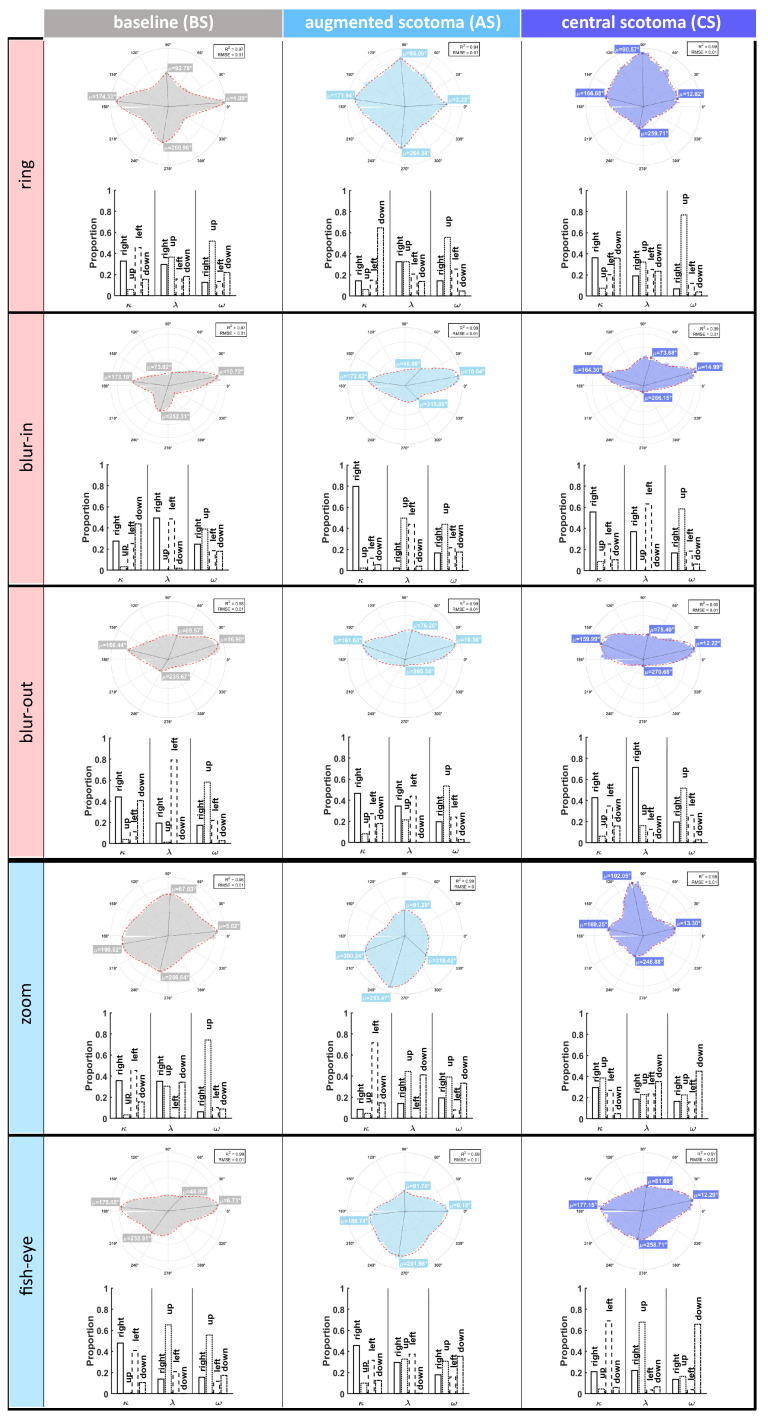
Polar histograms of gaze direction in respect to the target for all the five augmentations (cued monocular, red, and optical distortions binocular, blue) were tested across the three conditions (baseline, augmented scotoma, and central scotoma). The red dotted line represents the outcome of the fitted non-linear square model, with the R-squared and RMSE values plotted above each polar histogram. Additionally, the μ values for the right, up, left, and down directions are plotted. Beneath all polar histograms, an additional histogram representing the κ, λ, and ω parameters for each directionality is also presented.

For the ring augmentation (ring, Figure 5), during the baseline condition, the right and left directions had the highest concentration around the μ. During the monocular cue condition, the downward distribution was the highest one around its μ value with right and left decreasing. All through scotoma, the right and the down concentration parameters were equally the highest. The peakedness parameter remained mostly unaltered between the baseline, augmentation and scotoma conditions. The ω parameter evidenced a bias for the up direction during all three conditions.

During the baseline condition of the blur-in augmentation group (blur-in, Figure 5), the down direction had the highest concentration around the μ value. Once the blur-in assistance around the scotoma was introduced, the concentration value was the highest for the right directionality, while all the others decreased close to null values. After the assistance removal the concentration of the right distribution decreased. The peakedness parameter was the highest for the right and left direction during baseline, up and left during augmentation, and right and left again throughout scotoma. The bias parameter evidenced an upwards bias during baseline, augmentation and scotoma conditions.

For the blur-out augmentation group (blur-out, Figure 5), during baseline, the concentration value was the highest for the right and down direction distributions and right during augmentation and scotoma, with a slight decrease during the last condition. The λ parameter was the highest for the left direction during baseline, right and left during augmentation and only right during scotoma. An upwards bias during the baseline condition is observed, slightly decreasing with the augmentation and during the scotoma.

As to the distorted augmentations, for the zoom group (zoom, Figure 5), during the baseline condition, the right (*μ*: 5.02${}^{\circ }$) and the left (*μ*: 190.02${}^{\circ }$) direction distributions had the highest concentration. During the peripheral optical distortions, the left distribution concentration increased and became the highest, and during scotoma, right, up, and left distributions leveled up, while the down remained the lowest. All through baseline, the peakedness parameter was equally distributed around right, up, and down direction distributions. The up and down directions were the highest during augmentation, while all through scotoma, the down distribution has higher than right, up, and left. During baseline, an upwards bias was observed again. With the optical distortion, the tendency dropped and remained low during the scotoma condition. At the same time, the downwards direction bias increased both when the distortion was present around the scotoma and after its removal.

For the second distorted peripheral augmentation used, fish-eye (fish-eye, Figure 5), the right and left distributions had the highest concentration and low peakedness values during baseline. Throughout the augmentation condition, the concentration decreased, and the peakedness parameter increased for both. During the scotoma condition, the left distribution had the highest concentration for the left and the highest peakedness for the upwards one. Furthermore, for this group, an initial upwards bias was observed, and the bias decreased when the augmentation was introduced. In contrast, the downwards preference increased. During the scotoma condition, the upwards and downwards biases further reduced and intensified, respectively.

## 5. Discussion

Central vision is the most critical part of a person’s vision; thanks to it, it is possible to perceive an object of interest with very fine detail. Therefore, a visual disease such as scotoma that leads to central vision loss is very debilitating due to the impossibility of using such a central view. Those affected actively suppress their eyes from looking straight ahead and instead form a PRL in the intact peripheral vision.

Correctly allocating the PRL in the peripheral visual field is connected to maintaining a better or worse ability to see. Attention has been shown to play a crucial role in the influence of the PRL allocation [20,23,64,65].

The present study investigated the effects of a specific type of attention, salience [66,67], and of optically distorted augmentations over eccentric viewing. A VR pong game induced smooth pursuit during a target tracking task in participants with simulated scotoma. Five types of assistive peripheral stimuli, known to generate salience, were presented around the scotoma. Three were peripheral cues presented monocularly, and two induced peripheral distortions.

The study revealed that eccentric viewing was successful during the augmentation for all the peripheral monocular enhancements (red cued augmentations, the target was visible outside the scotoma area, Figure 4). Eccentric viewing was maintained for the scotoma condition after the cues were removed. Additionally, the baseline condition was significantly different from the scotoma condition after removing the augmentation. The fitting of the distribution of gaze relative to target revealed that monocular cued augmentations placed in the periphery could modify the distribution concentration and peakedness values around the four μ direction values. Changes in the concentration and peakedness meant that the distributions were less spread out whenever the κ values were high and less flattened around the μ value with high peakedness values. For the ring group, the concentration values of the right and left directions had more spread-out distributions when the assistance around the scotoma was used, while the down distribution less so. For the blur-in, the concentration value of the right direction was the least outspread one. The λ was more peaked around the up and left directions. As for the blur-out group, the augmentation condition spread out the down distribution. However, no trend emerged across groups, nor were any of the changes induced during the augmentations preserved during the scotoma condition.

Eccentric viewing was carried out successfully also during the distortions but not after removing them (blue distortion augmentation groups, target was not all the time visible outside the scotoma area, Figure 4). Same as for the cued monocular groups, the eyes behavior differed significantly between the baseline and the scotoma condition. However, eccentric viewing was not the strategy used. An efficient fixation frequency was observed during the scotoma condition (blue, Figure 3), indicating that the subjects could acknowledge the target by only directing their eyes at regular intervals to check for changes in the target. The distorted augmentations also induced changes in the distribution values of gaze in relation to target. Throughout the zoom augmentation condition the left distribution was less outspread while the up and down distributions were the most peaked ones. During the fish-eye augmentation the left distribution became more outspread and the peakedness increased. However, as observed for the previous cued monocular augmentations groups, the modifications induced did not have a tendency nor were further implemented during the scotoma condition.

It can be concluded that there is no trend in the distribution changes induced by either the cued or distortion augmentations used.

The weight parameter, an general indicator of a directional bias, evidenced an interesting behavior instead. Without simulated scotoma there was an upwards bias, and each peripheral assistant used further implemented the tendency or decayed it entirely.

The monocular augmentations preserved the bias during presentation and after being removed. The direction tendency further increased for two of the three monocular cues after the removal: ring, with the highest raise in such bias, and blur-in. The blur-out augmentation only preserved the tendency after the cue was removed. Nonetheless, the upwards bias remained the highest directional bias during scotoma simulation for all the groups where the monocular augmentations were used. Additionally, whenever a down bias was present, an increase in the upwards bias corresponded to a decrease in the downwards one during the ring and blur-in augmentation and scotoma conditions.

Distorted augmentations implemented the opposite: the upwards bias observed during the baseline condition decreased during zoom and the fish-eye. The fish-eye had a more substantial effect in reducing such tendency. Furthermore, to a decrease in the upwards direction corresponded an increase in the downwards one during the scotoma condition.

An upwards bias has been previously shown to be present during smooth pursuit [68]. The upwards directional bias implemented with peripheral monocular cues is consistent with previous literature. Attention stimulation mainly focuses the PRL on the lower part of the visual hemifield, where the highest attentional capability is allocated [23,65]. Additionally, an eccentric viewing behavior was preserved once monocular cues were removed. An eccentric viewing was not observed after the presentation of binocular distortions presentation. Additionally, the PRL was allocated in the higher part of the visual hemifield, which is known to have reduced attention capabilities compared to the lower one [65].

Given the current results, there is evidence that monocular cues could be better augmentations than binocular distortions. Eccentric viewing and directional bias are maintained after their removal, suggesting that subjects continue implementing the training they developed while having the scotoma and assistance. The established directional bias was the most efficient since it is where the highest attentional capability is. Once removed, one monocular cue, the ring, increased the directional bias even more so than the other monocular cues. It can be concluded that monocular ring augmentation might employ better attentional resources for eccentric viewing training.

Given the pilot nature of the study, additional studies with more participants are needed to confirm the advanced hypothesis. A further limitation to the study was the HTC Vive Pro Eye. As it was previously observed [38], an error of 1${}^{\circ }$ limited the rendering of a 6${}^{\circ }$ scotoma radii simulation when using the HTC Vive Pro Eye. The color change task provided the information that there was a 1${}^{\circ }$ error in the scotoma radii rendering. As previously [38], a sudden increase in the positive and a decrease in the negative predictive values were observed for the cued augmentation groups when the eyes were ≤5${}^{\circ }$ away from the target. Additionally, fixation frequency increased above 2, when the eyes were ≤5${}^{\circ }$ away from the target, indicative of inefficient visual processing. For all the peripheral augmentations, only when the eyes were ≥5${}^{\circ }$ away from the target did fixation frequency decrease, suggesting the target was visible only when the eyes were 5${}^{\circ }$ away from the target and not 6${}^{\circ }$. Nonetheless, the identified error remained consistent across the present and previous study. Furthermore, as previously conducted, the error was considered and corrected for.

In general, our study showed that gaming can be used for stimulating eccentric viewing and that monocular cues presented in the periphery can implement the best-suited attention resources. The results obtained for the ring augmentation confirm previous findings [24] where an upwards gaze direction strategy was also observed during and after removing a monocular peripheral concentric ring around a simulated scotoma. These results are encouraging for future patient applications where the VR pong game could be extended with multiple sessions of the augmentation condition applied to train eccentric viewing. Previous research showed that training periods could be extended up to 2.6 h since the duration of the training can play an important role in developing a PRL [69]. The effects seem to last up to 25 months [70]. Additionally, the longer the training period, the more prolonged the effect induced by the exercise will be. Nonetheless, 1 h long training sessions for a one-week-long period gave satisfactory results [71], suggesting a good starting point for future studies.

On the other hand, caution should be used for those studies implementing augmentations with peripheral distortions [22,72,73,74,75,76,77]. This is the first study to investigate the effects these assistive aids have on eccentric viewing behavior and results showed how peripheral distortions could negatively alter eccentric viewing and attentional resource allocation.

## 6. Conclusions

For the first time, the eccentric gaze behavior of normally sighted participants presented with monocular cues and binocular distorted peripheral augmentations around a simulated scotoma was investigated.

The study evidenced that monocular cues maintain eccentric viewing and better allocate attentional resources even after removal. The results confirmed previous results, encouraging the study’s application to patients to train eccentric viewing. Additional training sessions will need to be utilized for future patient applicability to induce a more long-term bias. The gaming and mixed reality nature of the training offers a perfect combination of engagement, learning facilitation, and frequent use motivation to suggest a successful usage of the system as a future eccentric viewing training tool. Binocular distortions in the periphery, on the contrary, canceled out both attentional resources and eccentric viewing. The negative impact on eccentric viewing behavior is to be considered for studies currently or from this point forward that will want to apply distortions as visual aids to patients.

## Data Availability

Data are available in figshare at the following doi: https://doi.org/10.6084/m9.figshare.20338911, accessed on 19 July 2022.
